# Opioid use disorder in Germany: healthcare costs of patients in opioid maintenance treatment

**DOI:** 10.1186/s13011-019-0247-9

**Published:** 2019-12-16

**Authors:** Jens Reimer, Tobias Vogelmann, Daniel Trümper, Norbert Scherbaum

**Affiliations:** 1 Center for Interdisciplinary Addiction Research, Dept. of Psychiatry and Psychotherapy, University Medical Center Hamburg-Eppendorf, Hamburg, Germany; 2 Center for Psychosocial Medicine, Health North, Hospital Group Bremen, Bremen, Germany; 3 LinkCare GmbH, Stuttgart, Germany; 4 Former: Indivior Deutschland GmbH, Mannheim, Germany; 5 LVR-Hospital Essen, Department of Addictive Behaviour and Addiction Medicine, Medical Faculty, University of Duisburg-Essen, Essen, Germany

**Keywords:** Opioid use disorder, Opioid maintenance therapy, Buprenorphine, Levomethadone, Methadone, Cost of illness

## Abstract

**Background:**

Opioid Use Disorder (OUD) is a substance use disorder with a chronic course associated with comorbid mental and somatic disorders, a high burden of psychosocial problems and opioid maintenance treatment (OMT) as a standard treatment. In the US, OUD imposes a significant economic burden on society, with annual societal costs estimated at over 55 billion dollars. Surprisingly, in Europe and especially in Germany, there is currently no detailed information on the healthcare costs of patients with OUD. The goal of the present research is to gather cost information about OUD patients in OMT with a focus on maintenance medication and relapses.

**Methods:**

We analysed health claims data of four million persons covered by statutory health insurance in Germany, applying a cost-of-illness approach and aimed at examining the direct costs of OMT patients in Germany. Patients with an ICD-10 code F11.2 and at least one claim of an OMT medication were stratified into the treatment groups buprenorphine, methadone or levomethadone, based on the first prescription in each of the follow-up years. Costs were stratified for years with and without relapses. Group comparisons were performed with ANOVA.

**Results:**

We analysed 3165 patient years, the total annual sickness funds costs were on average 7470 € per year and patient. Comparing costs of levomethadone (8400 €, SD: 11,080 €), methadone (7090 €, SD: 10,900 €) and buprenorphine (6670 €, SD: 7430 €) revealed significant lower costs of buprenorphine compared to levomethadone (*p* < 0.0001). In years with relapses, costs were higher than in years without relapses (8178 € vs 7409 €; SD: 11,622, resp. 10,378 €). In years with relapses, hospital costs were the major cost driver.

**Conclusions:**

The present study shows the costs of OUD patients in OMT for the first time with a German dataset. Healthcare costs for patients with an OUD in OMT are associated with more than two times the cost of an average German patients. Preventing relapses might have significant impact on costs. Patients in different OMT were dissimilar which may have affected the cost differences.

## Background

Opioid use disorder (OUD) is a substance use disorder characterized by compulsive opioid use, typically resulting in the development of tolerance and withdrawal after stopping the intake of the drug. Currently, there are several options available for the treatment of OUD, including behavioral therapies, opioid maintenance therapy using full opioid agonists (e.g. methadone), partial μ-opioid agonists (buprenorphine) or medication assisted treatment with opioid antagonists (naltrexone).

OUD imposes a significant economic burden on society in the US, with annual societal costs estimated between 55 billion dollars in 2007 [1] and 78.5 billion dollars in 2013 [2]. The majority of these costs are attributable to lost work productivity (46%) and healthcare costs (45%), with criminal justice costs (9%) making up the balance [1]. Florence et al. attribute 4% of 2013 costs to substance abuse treatment, 33% to healthcare, and 26% to lost productivity for nonfatal cases [2]. The remaining costs comprise lost productivity and healthcare for fatal cases (27%) as well as costs for criminal justice (10%). An additional burden of OUD are increased healthcare costs due to comorbid somatic diseases and mental disorders.

In a previous study, based on pharmacy and medical claims of 16 self-insured employer health plans in the US in the period of 1998 to 2002, opioid users were compared to a control group of non-abusers. Among these insured individuals, opioid abusers had higher rates of medical and pharmacy utilization and an increased number of comorbidities including poisoning, hepatitis, psychiatric illnesses, and pancreatitis. Healthcare expenditure for these individuals may have been as much as 8 times the total as non-drug users [3].

In Europe, and especially in Germany, there are currently no detailed information about healthcare costs of OUD patients. Available research with regards to Germany, the Netherlands and Switzerland, that investigated the costs of treatment with heroin and methadone, was based on calculations using various assumptions. Due to the lack of data availability, it mainly used administrative costs, such as diagnosis related groups (DRG) costs or listed pharmaceutical prices [4].

Therefore, to analyze healthcare costs of OUD patients in OMT, we designed a retrospective cost analysis in the German healthcare system from the statutory health insurance’s (SHI) perspective. The objective of the present study was to examine the overall healthcare costs of OUD patients in OMT using four cost categories: inpatient costs, outpatient costs, pharmacy costs and other costs that included sick pay, aids and remedies, and other medical services.

In Germany, there are currently 78,500 OUD patients in OMT [5]. Methadone, levomethadone and buprenorphine are the most commonly used treatments in OMT [5]. OMT can prevent relapses to illegally acquired opioids [6]. Concerning clinical evidence, it seems that buprenorphine in flexible doses adjusted to patients’ need is less effective than methadone (including levomethadone) in terms of treatment retention. For patients retained in treatment, there is no difference in suppression of illicit opioid use as measured by urinalysis or self-report. There are no differences between medium-dose buprenorphine (7–15 mg) and medium-dose methadone (40–85 mg) as to retention or suppression of illicit opioid use. Similarly, there are no differences between high-dose buprenorphine (≥ 16 mg) and high-dose methadone (≥ 85 mg) as to retention or suppression of self-reported opioid use [7]. In terms of mortality risks, it seems that buprenorphine could be more effective than methadone in reducing mortality, especially from overdose [8, 9].

Up to now, there are mixed findings regarding healthcare costs of different maintenance treatments: A systematic review indicates that pure pharmaceutical costs for methadone are somewhat less costly than buprenorphine [10]. However, previous research showed that although medication costs of buprenorphine might be higher than those of methadone, the overall healthcare costs including hospital and transportation costs may actually be lower [11].

Taken together, the present study addresses the research gap of assessing the overall healthcare costs of patients treated with common opioid maintenance treatments Germany and thus provides a picture of the current cost situation of OMT treated OUD patients in Germany.

## Methods

### Study design and data source

This longitudinal nested case-control cohort study was based on an anonymized German health claims database, including 4 million insureds from 8 German statutory health insurances, that provided a research database that could be used for this study. The dataset included a 5% sample of overall German population covered by statutory health insurances from January 1, 2010 to December 31, 2015. All patients in the database used for this analysis had complete data across all years of the analysis (2010–2015). Patients with incomplete yearly follow-up data were excluded unless they died within the study period and were in the dataset until death.

The dataset contained information on patients’ medical in- and outpatient claims, prescribed and delivered pharmaceuticals and insurance eligibility information. The whole study design was predefined by a detailed analysis protocol following the recommendation of the German society for epidemiology [12].

### Inclusion criteria

Patients with OUD were identified using the International Classification of Diseases, 10th Edition German Modification (ICD-10-GM) code F11.2 (mental and behavioral disorders due to use of opioids, dependence syndrome) in outpatient and/or inpatient care data in any of the quarters in the identification period (Quarter 01/2010 through 04/2011). For inpatient data, primary OUD discharge diagnoses as well as secondary diagnoses were considered. Outpatient physicians are required to designate their diagnoses as one of the following categories: validated, suspected, symptomless condition and excluded. A diagnoses becomes “validated” when it is established beyond doubt, e.g. by a lab test, a specialists’ assessment or the physicians’ own conclusions [13]. For the current analysis, only validated diagnoses were considered. The *index year* for each patient was defined as year when the first diagnosis of F11.2 was coded in 2010 or 2011. Although the ICD-10 code for OUD provides no information on the underlying opioid, recent data show that heroin use still accounts for the majority of new opioid-related treatment demands in Europe with around 80% of all patients with OUD [14]. Furthermore, patients needed at least one claim for OMT in the same quarter alongside one OUD diagnosis to be included in the study.

Other diagnoses than OUD were not included in the current analysis, because OMT is by law only available to patients with opioid dependence, not with other opioid abuse diagnoses [15].

### Exclusion criteria

Patients who were prescribed dihydrocodeine, diamorphine, or morphine between 2010 and 2015 were excluded from analyses. Moreover, patients who were younger than 18 years at their first OUD diagnoses in the inclusion period were excluded as they receive abstinence-oriented treatment, not OMT [16, 17]. Patients with no OMT in a year were excluded from cost analysis in the respective year, unless they had a *relapse* in the 12-months prior (see section Relapses for details). This was done because this study aimed to examine the healthcare costs of patients within OMT. Costs of OUD patients not in OMT were supposed to mainly occur within sectors outside of the health insurances and therefore not included in the database (e.g. drug counselling or controlled heroin use, both financed by municipalities in Germany or prison stays financed by federal government).

### Medical variables

The dataset included ICD-10-GM codes for all diagnoses given by inpatient or outpatient physicians that were analyzed in each quarter of the inclusion period. Comorbidities were assessed when assigned by a general practitioner or a specialist physician if the ICD-10-GM code was coded as “validated”: depression (F32X, F33X), personality disorder (F6X), anxiety (F40X F41.0, F41.1, F41.2), sleeping disorder (F51X), chronic hepatitis C (B18.2), chronic obstructive pulmonary disease (COPD, J44X), HIV/AIDS (B2X) and cardiovascular diseases (I20X-I25X).

### Medication variables

The dataset included Anatomical Therapeutic Chemical Classification (ATC) and pharmaceutical central numbers (PZN) for outpatient drugs prescribed and delivered.

OMT medications delivered to a physician’s office for supervised, office-based OMT were analyzed using the ATC codes for methadone, levomethadone, or buprenorphine respectively. OMT medication prescribed for take-home treatment was identified by pharmaceutical central numbers (PZN) from German auxiliary tax (*Hilfstaxe*) [18].

Table 1 shows the codes used to identify OMT in the different treatment groups.

**Table 1:** Prescriptions of OMT included in the analyses

	ATC-Codes	Pharmaceutical central numbers (PZN)
Treatment group methadone	N07 BC02	09999086
Treatment group levomethadone	N07 BC05	02567107
Treatment group buprenorphine	N07 BC01, N07 BC21, N07 BC51	02567113, 02567136

Patients were stratified in OMT groups with buprenorphine, methadone or levomethadone, based on the first prescription in each follow-up year. Patients could switch treatment groups in each follow-up year, according to their first prescription in each year, assessed in 12-month periods starting with each patient’s earliest relevant drug claim.

### Relapses

The definition of relapses was based on a previous longitudinal cohort study [19] and summarized three sorts of relapses: Firstly, relapse was defined as no OMT treatment for more than three months and returning to OMT thereafter (treatment interruption). Secondly, hospital stays with an acute intoxication defined via ICD-10 code F11.0 (Mental and behavioural disorders due to use of opioids: Acute intoxication) as main or secondary diagnosis were counted as relapse. Thirdly, death was derived from SHI data on the reasons of insurance termination. Reason of death was not included in the database, thus death of all causes was counted as relapse.

For attributing patients’ years with and without relapses, hospital stays, and treatment interruptions were counted in the year they started, i.e. hospital stays were counted in the year of the admission stay and treatment interruptions on the first day without OMT medication.

### Healthcare costs

Annual costs from the statutory health insurances database were assessed for each follow-up year and for four different resource use categories: inpatient care (hospital), outpatient care (office, also including outpatient psychotherapy), prescribed pharmaceuticals (outpatient setting only), and other costs that included sick pay, medical aids (e.g. wheelchairs) and remedies (e.g. physical therapy). Total costs were calculated as sum of the four resource use categories. Furthermore, costs were analyzed stratified for years with and without relapses.

### Statistical analysis

Variables were analyzed descriptively using frequencies and percentages for categorical variables and means with standard deviations for continuous variables. Group comparisons at baseline were performed using Chi-Squared tests and for categorial and t-tests for continuous variables. Group comparisons between different treatment groups were performed with analysis of variance (ANOVA) and least significant difference (LSD) post hoc tests. A *p*-value of *p* < 0.05 was used in all statistical tests for determining statistical significance. We used the software MS Excel 2016 and SAS 9.4, SAS Institute, Cary (North Carolina), USA for statistical analyses.

## Results

### Baseline characteristics

Overall, 996 patients fulfilled the inclusion criteria, 41 of the patients were excluded for being under 18 years old. The remaining 955 patients had a mean age of 37.6 years (standard deviation (SD) = 8.5). For these 955 patients, a total of 3165 patient years in the follow-up period could be observed. In the index year, 350 patients were treated with buprenorphine, 336 with methadone, and 269 patients with levomethadone (see Table 2).

**Table 2:** Descriptive statistics of the sample population in index year and in following follow-up years

Study year	Index Year *N* (%)	Follow-up 1 *N* (%)	Follow-up 2 *N* (%)	Follow-up 3 *N* (%)	Follow-up 4 *N* (%)	Patient years in Follow-up period
*N*	955	841	804	793	727	3165
OMT with bupren-orphine (%)	350 (36.6)	302 (35.9)	296 (36.8)	267 (33.6)	250 (34.4)	1115 (35.2)
OMT with levo-methadone	269 (28.2)	266 (31.6)	271 (33.7)	280 (35.3)	256 (35.2)	1073 (33.9)
OMT with methadone	336 (35.1)	273 (32.4)	237 (29.4)	246 (31.0)	221 (30.4)	977 (30.8)

Of 955 patients, 350 (36.66%) were treated with buprenorphine in the first year, 269 with levomethadone (28.16%) and 336 with methadone (35.18%). During follow-up, 23 patients (2.4%) died of any cause. From these 23 patients, one patient was treated with buprenorphine, 10 patients with levomethadone and 12 with methadone at the time of death.

Seven hundred twenty-seven patients (76.13%) stayed in OMT treatment during the whole four follow-up years, i.e. had at least one OMT prescription in each year. 59 patients (16.8%) changed from buprenorphine to methadone during the follow-up, 74 from buprenorphine to levomethadone (21.1%). From methadone, 30 patients (8.9%) changed to buprenorphine and 89 (26.4%) to levomethadone. 33 patients who were treated with levomethadone in the index prescription changed to buprenorphine (12.3%) and 78 changed to methadone (28.9%).

### Clinical characteristics

Across all treatment groups, the most frequent comorbidities at index date were depression (42.1%), chronic hepatitis c (38.2%) and personality disorder (15.0%) (see Table 3). Overall, there were 2782 patient years without relapses, 349 patient years with relapses as treatment interruption, 22 patient years with relevant hospital stays, and 12 patients deceased during the study period.

**Table 3:** Baseline characteristics

	Overall (*N* = 955)	buprenorphine (*N* = 350)	methadone (*N* = 336)	levomethadone (*N* = 269)	*p*-value
Mean Age (SD)	33.89 (8.3)	33.56 (8.5)	34.14 (8.2)	34.01 (9.1)	0.4681
Male (%)	681 (71.3)	258 (73.7)	243 (72.3)	180 (66.9)	0.1575
Depression	402 (42.1)	156 (44.6)	129 (38.4)	117 (43.5)	0.2247
Personality Disorder	143 (15.0)	39 (11.1)	54 (16.1)	50 (18.5)	0.0285
Anxiety	80 (8.4)	30 (8.6)	26 (7.7)	24 (8.9)	0.8607
Sleeping Disorder	118 (12.4)	47 (13.4)	25 (7.4)	46 (17.1)	0.0011
Chronic Hepatitis C	365 (38.2)	126 (36.0)	124 (36.9)	114 (42.4)	0.2292
COPD	63 (6.6)	20 (5.7)	29 (8.6)	14 (5.2)	0.1699
HIV/AIDS	19 (2.0)	3 (0.9)	11 (3.0)	5 (1.9)	0.0755
Cardiovascular Diseases	191 (20.0)	66 (18.9)	67 (19.9)	58 (21.56)	0.706
Alcohol Use Disorder	143 (14.9)	49 (14.0)	40 (11.9)	54 (20.1)	0.0162

### Healthcare costs

Based on the analysis of 3165 patient years, the total annually sickness funds costs for an OUD patient in OMT were on average 7470 € per year.

**Fig. 1:**
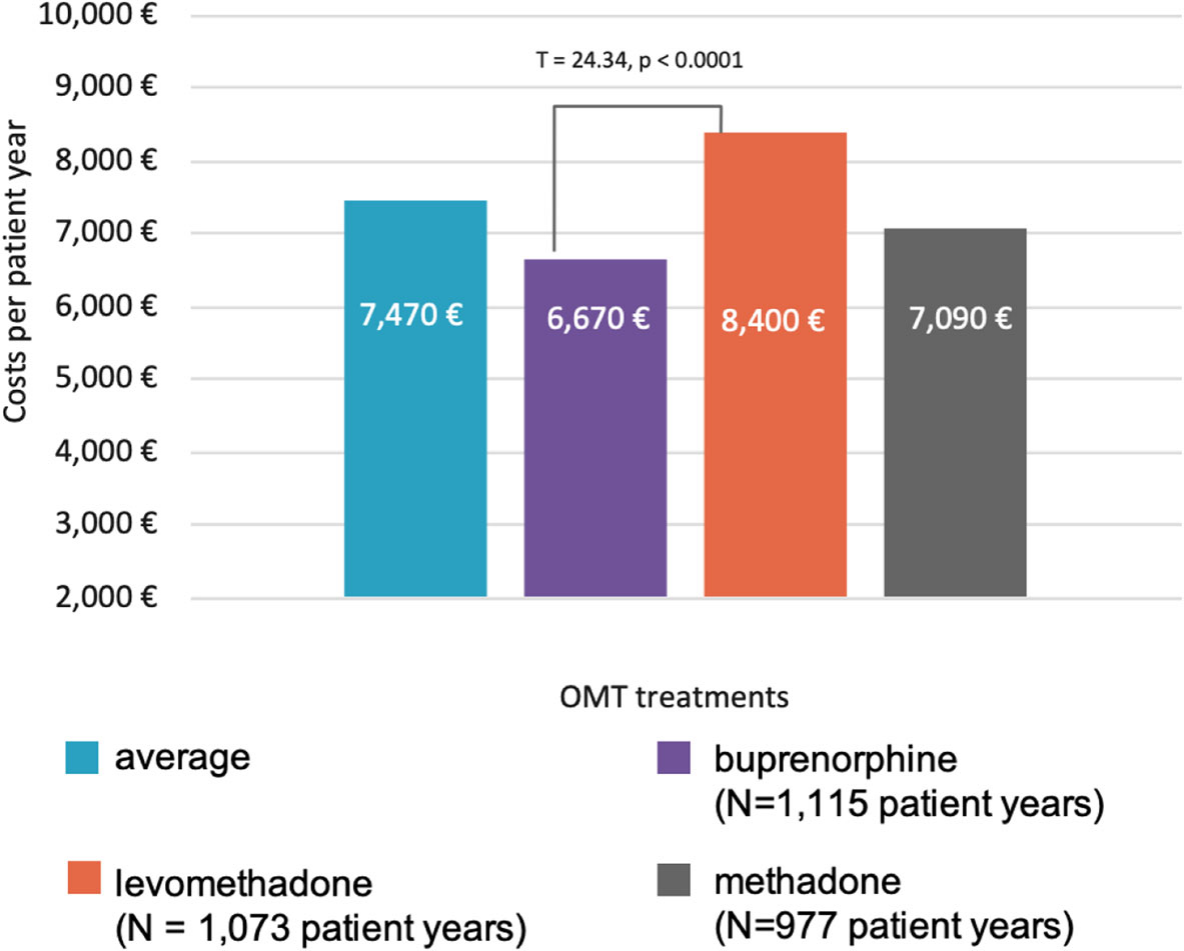
Cost comparison of three treatment groups, results of ANOVA

Comparing the 12-months costs of three treatment groups, levomethadone (8400 €, SD: 11,080 €), methadone (7090 €, SD: 10,900 €) and buprenorphine (6670 €, SD: 7430 €) revealed significant differences between the treatment groups (ANOVA, F = 14.19; *p* < 0.0001). Post hoc LSD test showed a significant difference between buprenorphine and levomethadone (t = 24.34; p < 0.0001). (see Fig. 1).

For buprenorphine treated patients, the costs were divided in four main cost categories as follows: inpatient costs: 35.01%; (2335 €) outpatient costs: 28.30% (1887 €); pharmacy costs: 32.41% (2162 €) and other costs: 4.28% (286 €). Costs of the methadone group consisted of inpatient costs accounting 38.83% (2753 €); outpatient costs: 25.02% (1774 €); pharmacy costs: 28.80% (2042 €) and other costs: 7.35% (521 €). And costs of the levomethadone group included 41.84% inpatient costs (3514 €), 24.39% outpatient costs (2049 €); 30.56% pharmacy costs (2567 €) and 3.21% other costs (269 €).

We compared treatment costs in years with and without relapses. Results revealed that costs in years with relapses were about 10% higher than in years without relapses with hospital costs as cost driver (8178 € vs 7409 €; SD: 11,622, resp. 10,378 €) (see Table 4).

**Table 4:** Cost breakdown in years with and without relapses

	Patient years with relapses	Patient years without relapses
Hospital costs in Euro (%)	4479.94 (54.78)	2990.90 (40.37)
Outpatient costs in Euro (%)	1765.64 (21.59)	1896.87 (25.60)
Pharmaceutical costs in Euro (%)	1294.35 (15.83)	2199.60 (29.69)
Other costs in Euro (%)	638.52 (7.81)	321.08 (4.33)
Costs (sum) in Euro	8178.45	7408.45

## Discussion

The present study examined overall healthcare costs associated with three different OMT in Germany. The average patient insured via German statutory health insurances induces healthcare costs of around 3034 € per year, a value obtained by the risk adjustment scheme of statutory health insurances [20]. We showed that healthcare costs for patients with an OUD in OMT are more than double these costs of an average German patient (7470 € versus 3034 €). These healthcare costs should be interpreted as total costs for treating patients with OUD in OMT and should not be interpreted as excess costs of OMT. Also, this number represents the average healthcare costs of patients either starting on OMT or continuing a prior course of therapy.

OUD is thus also associated with higher healthcare costs compared to widespread diseases like diabetes mellitus or depression with mean annual costs of 4949 € [21] and of 3638 €, respectively [22]. Costs per year are comparable to patients of chronic non-cancer pain patients in a long-term opioid therapy, where annual costs are around 7603 € [23]. All cited studies used comparable German SHI claims databases and assessed total healthcare costs, not excess costs, with the same methodological approach used in the current paper.

Extrapolated to the total number of 78,500 OMT patients in Germany [5], costs per year can be estimated to be around 588.4 million € in Germany. Again, these numbers represent overall treatment costs of OMT patients and not costs of OMT treatment per se. The real overall treatment costs are likely to be even higher than this extrapolation, for healthcare costs for privately insured OUD patients might be higher than for patients insured in SHI.

Overall costs for patients in buprenorphine treatment were lower than for those in methadone and levomethadone treatment. These results are in line with a previous study showing cost effectiveness of buprenorphine treatment in case different cost elements are considered [24].

The reason for this could be that buprenorphine can safely be dispensed as take-home doses earlier on in treatment if the patient is at low risk for diversion and can thus be dispensed with less effort [25]. As take-home treatment is around 30% less costly than office-based OMT in Germany [26], this could be one factor for lower costs of patients in buprenorphine based OMT. This would be consistent with the shown lower pharmaceutical and outpatient costs of buprenorphine treated compared to levomethadone treated patients. However, also dissimilarities within the different patient groups may have affected the cost differences, since the groups differed in occurance of sleeping disorder and personality disorder at baseline. Frequencies of other diseases associated with high treatment costs such as hepatits C [27] did not differ at baseline.

Costs in years with relapse were 767 € (around 10%) above years without relapses, indicating that preventing relapses might have positive effects on healthcare costs. This is in line with previous research that found associations between relapses and higher healthcare costs in other substance abuse patients: For alcohol addicted patients it was shown that patient groups with a lower risk of relapse also had lower healthcare costs [28].

### Strengths and limitations

The strength of this study is that it used a large sample of the German statutory health insurances to assess healthcare costs directly from a database with health insurance claims data. To our best knowledge, this is the first study to examine costs of patients in OMT in a major European country.

The limitations are as follows:

a) The statutory health insurances database uses billing data which are designed for invoices and not for research activities. Therefore, typically not all necessary information is available, or it has limited quality.

b) Persons entitled to other healthcare funds (e.g. members of the police), but also imprisoned patients are not covered by present data. About 10% of German population are members of private health insurance companies. There are differences between insureds of statutory and private health insurance companies in their socioeconomic status and health (care) behavior [29]. For this reason, the study sample is not completely representative for the general German population in terms of socioeconomic status.

c) We assume that total costs for patients without OMT are higher than indicated by present data because of social costs that are not reflected by statutory health insurances. Previous research [30] pointed out that drug abuse leads to more crime and lower employment. These social costs are not covered by health insurances and were not included in the present study. Moreover, a recent study shows that about one third of overall OUD costs in the US are due to substance abuse treatment costs and about one quarter represent the costs associated with public sector in healthcare, substance abuse treatment, and criminal justice costs [2].

d) There was no randomized assignment to the three drug groups. There is evidence that severely affected patients are more likely to receive methadone than buprenorphine [31, 32]. We did not control for this possible confounder, therefore such dissimilarities may have affected the cost differences.

e) Since the cause of death was not included in the database, death cannot directly be attributed to OUD. The assumption, that every death is counted as a relapse therefore overestimated the number of relapses. Since only 2.4% of the study population died within follow-up, this should, however, only have little influence on the results.

## Conclusions

In this study we analysed the annual healthcare costs of patients with OUD in opioid maintenance therapy from the perspective of the statutory health insurance in Germany. We showed that costs attributed to patients with an OUD and OMT are more than double the cost of an average patient in Germany. The present study presented costs of OUD for the first time with a large German dataset, comparing patients with different OMT. In sum, we found overall costs for buprenorphine treatment were lower than those for methadone and levomethadone treatment, although the reasons for this need further investigation. Furthermore, preventing relapses might have significant impact on reduction of costs. Which risk groups should be assessed and which OMT and dosages should be used for such relapse prevention, requires further research beyond the scope of this paper.

## Data Availability

The study was based on data from the statutory health insurances, which are protected by special data protection law. Restrictions apply to the availability of these data, which were used under license for the current study, and so are not publicly available. Data can be requested from the authors with permission of the involved statutory health insurances.

## Abbreviations

ANOVA: Analysis of variance
ATC: Anatomical therapeutic chemical classification
COPD: Chronic obstructive pulmonary disease
ICD-10-GM: International classification of diseases, 10th edition German modification
LSD: Least significant difference
OMT: Opioid maintenance therapy
PZN: Pharmaceutical central numbers
SD: Standard deviation
SHI: Statutory health insurance
